# Vitamin K and D Status in Patients with Knee Osteoarthritis: An Analytical Cross-sectional Study

**DOI:** 10.31138/mjr.32.4.350

**Published:** 2021-12-27

**Authors:** Alireza Askari, Mohammad Ariya, Sayed Hossain Davoodi, Hadi Raisi Shahraki, Elham Ehrampoosh, Reza Homayounfar

**Affiliations:** 1 Bone and Joint Reconstruction Research Center, Shafa Orthopedic Hospital, Iran University of Medical Sciences, Tehran, Iran,; 2 Noncommunicable Diseases Research Center, Fasa University of Medical Sciences, Fasa, Iran,; 3 National Nutrition and Food Technology Research Institute, Faculty of Nutrition Sciences and Food Technology, Shahid Beheshti University of Medical Sciences, Tehran, Iran,; 4 Department of Epidemiology and Biostatistics, Faculty of Health, Shahrekord University of Medical Sciences, Shahrekord, Iran

**Keywords:** osteoarthritis, vitamin K, vitamin D, pain, vitamin deficiency

## Abstract

Knee osteoarthritis (OA) is the most common form of arthritis and it has been known as the main factor of lower limb disability. The aim of the study is evaluating the level of vitamin K and D status as well as determining cut-off point of these vitamins for predicting knee OA and also pain severity in these patients. In this analytical cross-sectional study, participation included knee OA patients and individuals without it who referred to Fasa Osteoarthritis Clinic, Iran. OA was diagnosed according to criteria based on Kellgren and Lawrence rating and the WOMAC score was used for pain evaluation. Data analysis was performed in SPSS version 18 (P> 0.05). In our study, 150 knee OA patients and 300 individuals without it, were participated. The mean of vitamin K and D in OA patients was significantly lower than the group without it (P<0.001). Furthermore, logistic regression showed that after adjustment, each unit decrease in vitamin D and K level leads to an increase the odds ratio of OA to 0.67 and 0.002 times respectively. In receiver operator characteristic (ROC) curve analysis, the cutoff-point for vitamin D and K was determined 12.68 and 0.87 nmol/L respectively. We also observed that although with a decreased level of two vitamins, the score of pain significantly increases, the only effective factors in pain score were disease status. These findings suggest that the deficiency of vitamin K and D is likely associated with a higher risk of OA.

## INTRODUCTION

Knee osteoarthritis (OA) is the most common form of arthritis. The prevalence of the disease has been estimated to be 27 million individuals among adults in the United States.^1^ The disease typically affects the knee and causes pain. Furthermore, it has been known as the main factor of lower limb disability among American adults.^2^ To date, only a few treatment methods with low efficiency and side effects have been presented and there is not any treatment to prevent its occurrence and prevention.^3^ The new findings show that this disease is likely associated with age, gender, genetics, trauma, and metabolic syndrome.^3–5^

It is probable that vitamin K may play a role in OA. According to the literature, elderly people suffer from vitamin K deficiency in the United States and England.^6^ In a cross-sectional study, vitamin K deficiency was associated with the high incidence of hand OA^7^; furthermore, this deficiency was involved with knee OA progression and cartilage degradation.^3^ This vitamin plays an important role in mineralisation of bone and cartilage, and it is suggested that this effect is related to Gamma-carboxylation of Gla protein.^8^ Also, the result of a cross-sectional study support the hypothesis of vitamin K deficiency as a risk factor for knee OA.^9^

Vitamin D is associated with different aspects of cartilage and bone metabolism. Recent studies are rather controversial in terms of knee OA and vitamin D.^10^ There is a report about the relationship between vitamin D deficiency and high probability of incidence and progression of knee and pelvic OA.^11^ Besides, vitamin D serum levels deficiency will cause the degradation of joint space and increased osteophytes growth in knee OA.^12^ Alternatively, according to the randomised clinical trial study result, cartilage degradation has not been observed supportably in magnetic resonance imaging (MRI) of individuals with vitamin D deficiency.^13^ Furthermore, the results of a clinical trial study showed that vitamin D supplementations did not have any influence on joint cartilage degradation.^14^ Nevertheless, several studies on an individual with OA indicated the relationship between vitamin D deficiency and increased developments of symptoms and decreased joint space.^15^

Although there is evidence of the relationship between vitamin D or vitamin K deficiency and high risk of this disease, none of them has been studied two vitamins simultaneously. Besides, based on our knowledge there is only a few studies in the Middle East and Iranian population in this issue. So, regarding the controversial results of previous studies, the current study aimed to evaluate the level of vitamin K and D status as well as determine optimal cut-off-point for predicting OA and assess the relationship between the pain severity and level of two vitamins in these patients.

## MATERIAL AND METHODS

### Ethical consideration

The study protocol followed the Declaration of Helsinki guidelines and was approved by the Institutional Review Board (IRB) of Fasa University of Medical Sciences (Code: e-9314, 93129). Written consent was obtained from all the participants to enter the study.

### Study population

This study was an analytical cross-sectional study on knee OA patients registered in the Fasa Osteoarthritis Study known as FOAS. Registration has included all knee OA patients who have referred to Fasa hospital since 2013. Upon beginning the study, 150 patients with Knee OA had been recorded in the registration, all of whom participated in the present study. In addition, 300 individuals who came to the FOAS but did not present any signs of the knee OA and other kinds of OA, also took part in this research. It is important to note that knee OA was diagnosed according to criteria based on Kellgren and Lawrence (K&L) rating.^16^ Individuals with more than 1 point were considered as a patient.

Inclusion criteria for the group without OA were based on lack of any complaint about stiffness and roughness in the motion of knee joints and lack of any common problems arisen from any other types of OA which are detected in graphics (K & L= 0 point). Furthermore, people have an extreme change in diet, being on a diet in the last 2 and 3 months, taking blood fat/sugar lowering pills, participants younger than 18 years and older 65 years, and one of the illnesses affecting a patient’s metabolic status, such as cancer, liver disease and diabetes, were considered as exclusion criteria.

### Vitamin K and D Evaluation

Blood samples were collected from all individuals in the central laboratory of the university after 10 hours fasting. All blood samples were immediately centrifuged at 3,000 rpm for 10 minutes at 4°C and their serum was separated and classified and froze in −70 °c for less than 2 years and away from light and were immediately heated after removing from the freeze.

Phylloquinone is a biochemical criterion of vitamin K which remains stable under determined restoring condition and was assessed by the reversed-phase high-performance liquid chromatographic reaction, solid-phase chemical reduction, and fluorometric detection. Vitamin D (25-OH D3), also, was measured by the ELISA (Enzyme-linked immunosorbent assay) kit (Enzo Lifesciences, Farmingdale, New York).

### Evaluation of pain score

In this study, for evaluating pain, the Western Ontario and McMaster universities arthritis index score (WOMAC) was used. The index consists of 24 items that completed by self-administered and broadly was applied in the evaluation of hip and knee OA (17). Based on the answers given to the questionnaire, it is divided into 3 subscales: Stiffness (2 items), Pain (5 items) and Physical Function (17 items).^17^

### Statistical Methods

Descriptive statistics were reported for quantitative and qualitative variables as mean (SD) and number (%) respectively. Univariate analysis performed using chi-square or independent T-test. To assess the simultaneous effect of variables on OA disease, logistic regression was implemented, and linear regression was used to modelling effective factors on pain score. Due to the nature of highly correlated variables in the current study, using traditional variable selection techniques in regression modelling was not appropriate. Therefore, to avoid multicollinearity challenges, one of the best-penalized regression methods called smoothly clipped absolute deviation (SCAD) was implemented. SCAD performs simultaneous estimation and variable selection via a regulation parameter that estimates using k-fold cross-validation technique. To assess the classification accuracy of the SCAD regression models, probability of OA for each participant was estimated using the proposed model and optimal cut-off point was obtained using the receiver operator characteristic (ROC) curve analysis. All the statistical analysis was performed in ncvreg package in R 3.3.1 software or SPSS 18.0.

## RESULTS

In our study, 150 patients with knee OA and 300 people that referred to osteoarthritis clinic but did not present any signs of the knee OA and other kinds of OA, participated. Most of the participants with knee OA (83.2%) and without it (81.3%) were female and there was no significant difference between them (P=0.80). Moreover, compared to the group without OA, knee OA patients had a higher level of body mass index (BMI) and waist circumference that was statistically significant (P<0.001) (**Table 1**).

**Table 1.** Comparison of the characteristics of the subjects between the two groups.

	Group without OA (n=300)	Knee OA patients (n=150)	p-value^*^
Age (years)	56.5 (9.1)	54.3 (9.1)	0.02
Waist circumference (cm)	86.8 (4.5)	91.7 (4.9)	<0.001
BMI (kg/m^2^)	26.5 (2.7)	28.8 (2.5)	<0.001
Vitamin D level (nmol/L)	66.2 (21.9)	18.0 (7.3)	<0.001
Vitamin K level (nmol/L)	3.5 (0.8)	1.1 (0.4)	<0.001
Pain score	5.6 (2.6)	15.5 (3.2)	<0.001

BMI: body mass index; OA: osteoarthritis.

* Independent T-test.

Mean (SD) of vitamin D level in the people without OA was 66.2 (21.9) nmol/L and in knee OA patients was 18.0 (7.3) nmol/L which was statistically significant (P<0.001). Independent T-test also showed that two groups were very different in term of vitamin K level and the mean of vitamin K in knee OA patients was significantly lower than participants in the group without OA (1.1 [0.4] versus 3.5 [0.8], P<0.001). Two groups were different also in pain score and as expected, pain levels were significantly higher in OA patients (**Table 1**).

In **Table 2**, using 10-fold cross-validation technique, the regulation parameter was estimated 0.006 and 0.01 for the first and second SCAD logistic regression respectively. In the first model, SCAD logistic regression showed that after sex, age and BMI adjustment, each unit decrease in vitamin D level leads to an increase in the odds ratio of knee OA to 0.67 times. Also, the probability of OA for each case was calculated using SCAD model as follow:

**Table 2.** Relationship of knee osteoarthritis with vitamin D and K status.

Variable	Model 1			Model 2		
	Coef	OR^*^	95 % CI	Coef	OR^*^	95 % CI
Sex	0.0	1.00	0.10–9.44	0.0	1.00	1.00–1.00
Age (years)	−0.20	0.82	0.64–1.04	0.0	1.00	1.00-1.00
BMI (kg/m^2^)	1.31	3.71	1.27–10.81	0.34	1.40	1.02–1.94
Vitamin D level (nmol/L)	−0.406	0.67	0.57–0.78	-	-	-
Vitamin K level (nmol/L)	-	-	-	−6.23	0.002	0.00–0.06

BMI: body mass index; Coef: Coefficient; OR: odds ratio; CI: confidence interval.

* SCAD logistic regression.


$${\text{p}}_{\text{i}}=\frac{{\text{e}}^{-13.5-0.2*\text{age}+1.31*\text{BMI}-0.406*\text{vitamin}\;\;\text{D}\;\text{level}}}{1+{\text{e}}^{-13.5-0.2*\text{age}+1.31*\text{BMI}-0.406*\text{vitamin}\;\;\text{D}\;\text{level}}}$$

Optimal cut off point was determined as p=0.31 in receiver operating characteristic (ROC) analysis. The area under the ROC curve (AUC) of the proposed model was equal to 99.8% (99.0- 100) and its sensitivity and specificity was 99% and 97% respectively.

In the second model, a separate SCAD logistic regression was used to modelling an association between vitamin K level and OA disease. The proposed model revealed that after sex, age, and BMI adjustment, for each unit increase in vitamin K level, odds of OA decrease to 0.002 times. Moreover, the probability of OA for each case was calculated using the following model. In the model, optimal cut off point were determined as p=0.43 in ROC analysis and AUC of the proposed model was equal to 100% (99.2–100) so both sensitivity and specificity were 100%:

$${\text{p}}_{\text{i}}=\frac{{\text{e}}^{2.53+0.34*\text{BMI}-6.23*\text{vitamin}\;\;\text{K}\;\text{level}}}{1+{\text{e}}^{2.53+0.34*\text{BMI}-6.23*\text{vitamin}\;\;\text{K}\;\text{level}}}$$

**Figure 1.**
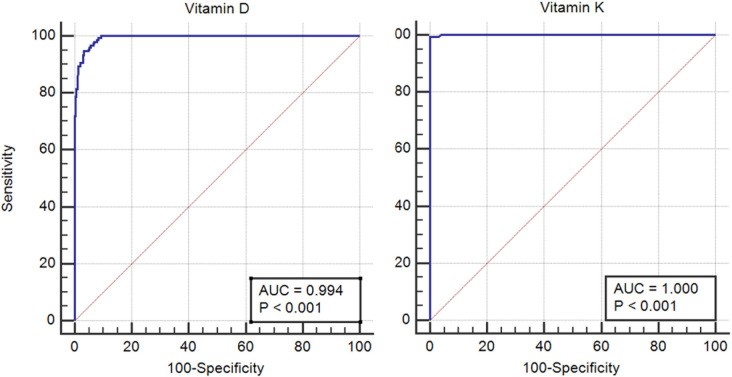
ROC curves for vitamin D and vitamin K for Osteoarthritis.

**Table 3** and **Figure 1** show the cut point levels for vitamin D and vitamin k concerning knee OA. According to this table, the optimum vitamin D cut-off point for predicting knee OA was defined as 12.68 nmol/L (Auc = 99.4%) and for vitamin K was 0.87 nmol/L (Auc=100%). These figures also indicate that the sensitivity and specificity of vitamin K for predicting risk of knee OA is higher than vitamin D.

**Table 3.** Receiver operating characteristic (ROC) curve analysis for defining the ideal Vitamin D and vitamin K cut-off point.

	Cut Point*	**Sensitivity**	**Specificity**	**AUC**	**PPV**	**NPV**	**PLR**	**NLR**
Vitamin D (nmol/L)	**12.68**	94.67	96.67	0.994	93.4	97.3	28.40	0.055
Vitamin K (nmol/L)	**0.87**	99.33	100	1.00	94.3	99.7	33.11	0.0069

AUC: Area under the curve; PPV: Positive predictive value; NPV: Negative predictive value; PLR: Positive likelihood ratio; NLR: Negative likelihood ratio.

* The optimum cut-off was defined as the point on the ROC curve with the maximum sensitivity + specificity (Youden method).

**Figure 2.**
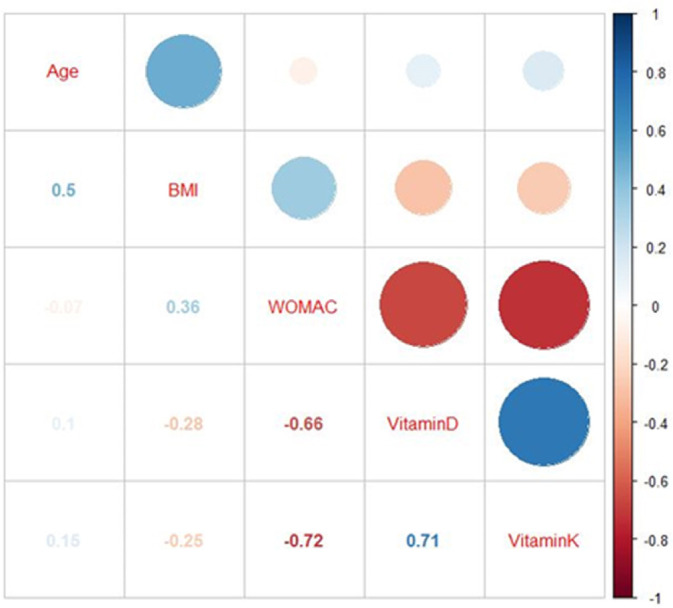
Pearson correlation between the studied variables. WOMAC: Western Ontario and McMaster universities arthritis index score.

Pearson correlation revealed that there was a strong negative correlation between pain score and vitamin D level (r=−0.66) which was statistically significant (P<0.001). Besides, a significant and negative correlation exists between pain score and vitamin K level (r=−0.72, P<0.001). Moreover, a positive correlation was observed between amounts of vitamin D and K (r=0.71) which was statistically significant (P<0.001) (**Figure 2**).

ANOVA analysis between pain score quartiles reveals that there are significant differences between both vitamin levels (**Table 4**). In this table, we reveal that with a decreased level of two vitamins, the score of pain significantly increases (P<0.001) and in the fourth quartile, we saw the highest level of pain and the lowest level of two vitamins simultaneously.

**Table 4.** ANOVA analysis of vitamin D and K in pain quartiles.

	Pain level quartiles (WOMAC score)				
	<= 5.16	5.17 – 7.15	7.16 – 12.83	12.84=<	P value
Vitamin D (nmol/L)	29.57 ± 10.05	29.09 ± 9.64	23.36 ± 13.41	8.21 ± 4.79	<0.001
Vitamin K (nmol/L)	1.56 ± 0.37	1.59 ± 0.38	1.27 ± 0.62	0.49 ± 0.28	<0.001

WOMAC: Western Ontario and McMaster universities arthritis index score.

SCAD linear regression revealed that after sex, age, and BMI adjustment, the only effective factors on pain score were disease status and level of vitamin D and K had no effect on pain score (**Table 5**). After adjusting variables, the mean score of pain in OA patients was 9.93 higher than others.

**Table 5.** Relation between pain severity and the variables studied.

Variable	Coefficient^*^	SE
Sex	0.0	0.45
Age (year)	0.0	0.38
BMI (kg/m^2^)	0.0	0.01
OA	9.93	0.06
Vitamin D level (nmol/L)	0.0	0.01
Vitamin K level (nmol/L)	0.0	0.11

* SCAD linear regression, adjusted R square= 0.59, BMI: body mass index; OA: osteoarthritis.

It is noteworthy to mention that the relationship between vitamin K and D levels and BMI was analysed and the results are shown in supplementary **Table 1**. The results of this table showed that regardless of BMI, the levels of vitamins K and D are significantly different between the two groups and probably do not accept a significant effect on BMI levels (P= <0.001).

## DISCUSSION

The result of the current study showed that the level of vitamin D and vitamin K in patients with OA is significantly lower than the group without it. These findings suggest that the deficiency of these two vitamins is likely associated with a higher risk of knee OA and the risk of knee OA development would rise if vitamin D and K decreased from 12.68 and 0.87 nmol/L, respectively, and the sensitivity and specificity of vitamin K for predicting the risk of knee OA is higher than vitamin D. Furthermore, according to the regression models, by decreasing each unit of vitamin D and vitamin K levels, the risk of disease increases about 0.67 and 0.002 times, respectively.

In this scope, the result of studies is controversial. The result of the present study is consistent with other studies which suggested that a low level of vitamin K or D plays a role in the increased risk of OA.^18,19^ In this way, Neogi et al. (2006) found that the greater incidence of knee OA is associated with low plasma phylloquinone concentration.^7^ Furthermore, in an elderly cohort study, Oka et al. (2009) showed that there is a significant relationship between food intakes of phylloquinone sources and decrease the incidence of OA.^9^ However, some study shows that vitamin D does not have an effect on the disease^20^ and also there is not any relationship between the supplementation intakes of vitamin K and OA risk.^21^

This controversial results could be related to the nature of conducted research or population under study.

The result of the present study showed that despite the significant negative relationship between the levels of vitamin K and D and knee OA incidence, the serum level of these two vitamins has no influence on the pain level which arise from this disease and after the adjusting for demographic variables like gender, age, and BMI, only OA disorder itself impacts on pain. However, the findings of the current study do not support those studies which indicated contradictory results. Accordingly, a study found that vitamin D deficiency is associated with knee OA pain^18^ and other indicated that the consumption of vitamin D supplement decreases the pain.^22^ It seems that we need further study in this regard.

The possible mechanisms in those studies which consider vitamin D as a factor for relieving pain indicate that the active form of vitamin D has anti-proliferative characteristic and prevent from the proliferation of inflammatory factor,^23^ therefore, it causes the modulation of knee pains (except those pains arise from the weight tolerance).^24^ On the other hand, another study showed that vitamin D supplementation modulates knee OA pain by strengthening quadriceps muscle.^25^ Finally, it can be claimed that the result of studies is extremely contradictory regarding the relation between vitamin K and D and OA pain, and more studies need to be conducted (particularly on RCTs) in this sense.

Several studies have recommended mechanisms based on how these two vitamins cause a decreased risk of OA. Accordingly, it has been identified that vitamin D absorb calcium in bones and vitamin K is involved in γ-carboxylation of glutamic acid residues in most of the proteins that depend on calcium.^26^ We know that vitamin K contributes as a major cofactor in γ-carboxylation of glutamate enzyme involving in γ-carboxylation of bone tissue proteins such as matrix Gla (gamma-carboxy glutamic) protein (MGP).^26^ Matrix Gla protein (MGP) is an extracellular matrix of a family of Gla protein-containing osteocalcin and the growth arrest-specific protein 6 (Gas6). Furthermore, Gas6 is associated with up-regulated of growth-arrested cells, and likely, it plays a protective role in apoptosis and other cellular stresses prevention.^27^ In this regard, studies reported that Gas6 is effective in promoting survival and cell replication.^28^ On the other hand, decreased Gas6 probably lead to low survival level of chondrocyte (cells inside the cartilage) in joint cartilage, which is considered as one of the important incidence factors of this disease.^8^

Additionally, since MGP is presented by proliferative and late hypertrophic chondrocytes,^29^ its mutation is responsible for occurring of Keutel syndrome in patients exposing to abnormal cartilage calcification.^30^ The studies which carried out on mice with MGP deficiency showed that MGP acts as an inhibitor of extracellular matrix calcification on the epiphyseal growth plate.^31^ According to these evidences, as an inhibitor of extracellular matrix calcification and a factor of cell survival and proliferation, vitamin K plays a predominant role in cartilage metabolism. Therefore, the permanent consumption of vitamin K in diet impacts on MGP and Gas6 which are dependent on this vitamin and improve OA pathogenesis by changing in the process of osteoporosis and cartilage degradation.^9^ Additionally, vitamin K itself copes with inflammatory cytokines which are contributed to osteoporosis.^32,33^ Therefore, it could be concluded that the plasma level of phylloquinone is reversely associated with knee OA incidence.

In the present study, the association was observed between the low level of vitamin D and the increased risk factor of OA. In this context, studies have suggested several mechanisms for the relationship between vitamin D and OA. First, although OA pathogenesis is not clear, recent studies found that the imbalance of subchondral bone may start this degenerative change.^34^ Vitamin D deficiency is likely associated with the degenerative change as the decreased serum level of 25-(OH) D result in accelerating osteoclastogenesis process, absorbing bone, and OA.^35^

The second mechanism is related to the relationship between vitamin and muscle weakness. The result of studies indicates that vitamin D supplementation reduces the fracture and falls risk, resulting in the positive influence of this vitamin on the muscular force, bone balance and health among the old population.^36^ These findings mirror those of the other studies that have examined the individuals with knee OA.^37^ Likewise, these studies report that individuals with vitamin D deficiency encounter with more problem on mobility and do the daily activity than the group who has the normal level of this vitamin. This indicates that vitamin D deficiency is associated with muscle weakness, decreased quality of life, and OA intensification.^38^

Another mechanism considers OA treatment by vitamin D. Because vitamin D has receptors on chondrocyte, particularly in individuals who suffer from a deficiency, it has been suggested as a possible treatment of OA.^39^ Vitamin D can regulate metalloproteinase matrix by this receptor.^40^ Therefore, it can be claimed that vitamin D deficiency is involved with pain sensitivity, muscle weakness, decreased body strength, and low quality of life.^41^ However, some studies found that vitamin D does not have such influence,^42^ and studies such as that conducted by Salman et al. did not support the consumption of vitamin D supplementation for improving OA.^43^ The reason for this likely arises from the differences in amount or consumed supplements dose or the difference on the blood level of the amount of vitamin D in the individuals understudied. Therefore, it is recommended that more studies should be done to find a definite answer to this subject.

The last mechanism examines the relationship between vitamin D and calcium in OA, and regarding its role on calcium metabolism in the body, it points out that affecting by this disease and body vitamin D status is predictable which several epidemiologic studies have shown this relationship.^44^ Specifically, according to the studies, although vitamin D deficiency causes secondary hyperthyroidism and this lead to an increase in bone turnover and a reduction in BMD, if vitamin D deficiency and high Parathyroid hormone (PTH) occur simultaneously, the possible incidence of OA increases.^45^

As far as the writers of the studies know, few similar studies have been investigated in this issue in Iran and none of them had this sample size. Based on our knowledge, none of the previous studies have not been investigated two vitamins simultaneously. However, the study has some deficiencies and limitations. The analytical cross-sectional nature of the study is its main limitation which does not allow to a causal conclusion or reversal relationship. Therefore, it is recommended that this kind of studies should recur in terms of cohort and longitudinal studies to provide more attributable results. Although there was a registration system which minimises the possible biases in selection and examining the significant population out of the total considered region, lack of financial resources restricted the investigation of more variables to consider more metabolic and hormonal issues for the population of the study.

## CONCLUSION

Our study shows a significant relationship between reducing vitamins K and D serum levels with the risk of OA. Our observation also indicates that the likelihood of OA development rose significantly with decreasing the amount of vitamin D and K from the cut-off point level. We also conclude that despite the significant relationship between the levels of vitamin K and D and OA prevalence, after adjustment, the serum level of these two vitamins was not an influential factor on the pain level.

**Supplementary Table 1.** Vitamin K and D levels compare between Osteoarthritis and control groups in normal weight and overweight subjects.

BMI status		Vitamin D level	Vitamin K level
Normal (<25)	Control	30.70± 8.83	1.60± 0.40
	OA	7.01± 3.31	0.14± 0.06
P-value		<0.001	<0.001
Obese (>25)	Control	29.47± 10.22	1.60± 0.36
	OA	8.17± 3.29	0.51± 0.17
P-value		<0.001	<0.001

## DATA

The data that support the findings of this study are available on request from the corresponding author.
